# Micro-computed tomography for gunshot residue detection: A systematic review and meta-analysis of distance-dependent deposition patterns

**DOI:** 10.1007/s00414-026-03879-x

**Published:** 2026-06-22

**Authors:** Carlos Antonio Vicentin-Junior, Raíssa Bastos Vieira, Luciana Munhoz, Plauto Christopher Aranha Watanabe, Carlos Eduardo Palhares Machado, Paulo Ricardo Martins-Filho

**Affiliations:** 1 Present Address: Graduate Program in Pathology, Department of Pathology and Legal Medicine, Ribeirão Preto Medical School, University of São Paulo, USP, Av. Bandeirantes 3900, 14049-900 Ribeirão Preto, Brazil; 2 Department of Stomatology, School of Dentistry, University of São Paulo, São Paulo, SP Brazil; 3 Department of Stomatology, Public Oral Health, and Forensic Dentistry, Ribeirão Preto Dental School, University of São Paulo, Ribeirão Preto, Brazil; 4 National Institute of Criminalistics, Brazilian Federal Police, Distrito Federal, Brazil; 5 Investigative Pathology Laboratory, Federal University of Sergipe, Sergipe, Brazil

**Keywords:** Micro-computed tomography, X-ray microtomography, Radiodense particulates, Gunshot residue, Volumetric analysis, Methodological limitations

## Abstract

Micro-computed tomography (micro-CT) has emerged as a non-destructive technique for three-dimensional visualization of radiodense gunshot residue (GSR), yet its quantitative performance and variability have not been systematically evaluated. This systematic review and meta-analysis synthesized experimental evidence on micro-CT–based detection of inorganic GSR. Following PRISMA guidelines, six studies were included, of which four provided data for quantitative synthesis. Random-effects meta-analyses showed a progressive decline in detectable residue with increasing shooting distance, with pooled detection rates of 0.133% at 5 cm and 0.019% at 30 cm, accompanied by extreme between-study heterogeneity (I² > 99%). Quadratic mixed-effects meta-regression identified a significant nonlinear association between distance and detection, with a predicted maximum at approximately 17.79 cm, although distance explained only a small proportion of variability. Exploratory Gaussian modeling revealed marked substrate-dependent differences in detection profiles. Overall, micro-CT consistently enabled non-destructive three-dimensional localization and volumetric assessment of radiodense particles but showed limited capacity for standalone inferential applications, particularly shooting-distance estimation. These findings support its role as a complementary screening and mapping tool within forensic ballistic investigations while highlighting the need for standardized protocols and confirmatory compositional analyses. Clinical trial number: not applicable.

**Supplementary Information:**

The online version contains supplementary material available at 10.1007/s00414-026-03879-x.

## Introduction

Imaging technologies have become increasingly integrated into forensic sciences due to their ability to identify, visualize, and preserve two- and three-dimensional structures in a non-destructive manner [1–3]. Among these modalities, computed tomography (CT) has gained relevance by enabling volumetric reconstruction and supporting both qualitative and quantitative analyses while minimizing physical intervention on the specimen [4–8]. These attributes have positioned CT as a valuable adjunct to conventional forensic examinations. Nevertheless, its spatial resolution remains insufficient for the investigation of structures at the micrometric scales, thereby limiting its applicability to the analysis of microtraces [9].

Micro-computed tomography (micro-CT) has emerged as a high-resolution extension of conventional CT, operating on the same physical principles while providing substantially enhanced spatial resolution [10–12]. This advancement enables detailed three-dimensional reconstructions and precise volumetric and density measurements at the microscale. As a result, micro-CT offers a comprehensive spatial assessment of microtraces [9, 13], defined as minute traces that are not discernible without magnification or advanced imaging techniques [14, 15].

Within the spectrum of forensic microtraces, ballistic-related evidence warrants particular attention, given the persistently high global burden of firearm-related violence and homicide [16]. Among such evidence, gunshot residue (GSR) represents one of the most frequently examined trace materials. GSR consists of metallic and non-metallic microparticles generated during cartridge deflagration and firearm discharge, which may be deposited on the shooter, the victim, or surrounding surfaces [17, 18]. Currently, scanning electron microscopy coupled with energy-dispersive X-ray spectroscopy (SEM/EDS) is widely regarded as the gold standard for GSR analysis due to its high sensitivity and elemental specificity [19]. However, complementary analytical techniques, including colorimetric assays, Raman spectroscopy, time-of-flight secondary ion mass spectrometry [20, 21], and micro-CT [22] have been increasingly explored to overcome methodological limitations and expand interpretative frameworks.

The application of micro-CT to GSR analysis provides distinct advantages, particularly through non-destructive visualization of the three-dimensional spatial distribution of residue particles, as well as micrometric measurements of particle size, morphology, and density. These capabilities support a more refined understanding of the ballistic behavior of GSR across different substrates and contribute to inferential approaches related to shooting distance estimation [22]. Despite its theoretical and practical potential, the literature addressing the use of micro-CT in GSR analysis remains limited and methodologically heterogeneous.

Considering the current evidence, the present study aimed to systematically synthesize and critically appraise the available evidence regarding the application of micro-CT for the visualization, analysis, and characterization of gunshot residues. Additionally, it aimed to quantitatively evaluate the relationship between micro-CT–detectable residues and shooting distance, thereby clarifying the current evidentiary value of this technique within forensic ballistic investigations.

## Materials and methods

This systematic review was conducted in accordance with the Preferred Reporting Items for Systematic Reviews and Meta-Analyses (PRISMA) guidelines [23] and the Meta-Analysis of Observational Studies in Epidemiology (MOOSE) statements [24]. The study protocol was prospectively registered on the Open Science Framework (OSF; 10.17605/OSF.IO/VDUSW).

### Research question and eligibility criteria

This review aimed to determine whether micro-CT is applicable for the detection, characterization, and potential estimation of shooting distance based on GSR in biological and non-biological forensic samples.

Eligible studies were those employing micro-CT to investigate GSR in the aforementioned specimens, without restrictions regarding language or publication date. Reviews, editorials, expert opinions, conference abstracts, case reports, and very small case series (< 5 cases) were excluded. When multiple publications reported potentially overlapping experimental datasets, the study with the largest sample size or the most comprehensive data was retained.

Studies with limited sample sizes (5 ≤ *n* < 10 cases) were included exclusively in the qualitative synthesis to ensure maximal coverage of the available evidence, while acknowledging the inherent limitations associated with reduced statistical precision.

### Search strategy

A systematic literature search was conducted on PubMed, Web of Science, Scopus, Embase, Google Scholar, and Open Access Theses and Dissertations (OATD). For Google Scholar and OATD, the first 100 retrieved records were screened. Additionally, reference lists of all eligible studies were manually examined to identify further relevant publications. The searches were conducted on October 15, 2025, and updated on January 7, 2026.

The search strategy combined controlled vocabulary and free-text terms as follows: (“micro-CT” OR “microCT” OR “micro computed tomography” OR “micro-computed tomography” OR “X-ray microtomography” OR “microtomography” OR “computed micro-tomography” OR “computed microtomography”) AND (“gunshot residue” OR “GSR” OR “shot residue” OR “shotgun residue”).

### Study selection

Study selection was independently performed by two reviewers (C.A.V-J. and R.B.V.) using a two-stage screening process. Duplicate records were identified and removed using the Rayyan platform [25]. Titles and abstracts were subsequently screened, followed by full-text assessment of potentially eligible studies. Discrepancies were resolved by consensus or, when necessary, by consultation with a third reviewer (L.M.).

### Data extraction

Data extraction was conducted independently by two reviewers (C.A.V-J. and R.B.V.) using a standardized form. Extracted information included: authorship, year of publication, country, sample size, sex and age range (when applicable), firearm type, caliber, projectile characteristics, firing setup, gunshots per distance, firing distances, and outcomes. Detailed information on micro-CT acquisition and processing parameters was also collected, including scanner model, voltage, current, filter, voxel size, field of view, rotation parameters, scan duration, volume of interest, reconstruction and analysis software, Hounsfield unit calibration, and GSR segmentation thresholds. All outcomes related to micro-CT performance were recorded.

### Risk of bias assessment

The methodological quality and risk of bias of included studies were assessed independently by two. reviewers (C.A.V-J. and R.B.V.) using the Joanna Briggs Institute (JBI) Critical Appraisal Checklist for Analytical Cross-Sectional Studies (https://jbi.global/critical-appraisal-tools). Although most included investigations were experimental or bench-based ballistic studies, this tool was selected to provide a structured and transparent appraisal of key methodological domains.

The assessment addressed eight criteria: clarity of inclusion criteria; adequacy of sample and setting description; validity and reliability of exposure measurement; objectivity of outcome assessment; identification of confounding factors; strategies to address confounding; validity and reliability of outcome measurement; and appropriateness of statistical analysis. Each item was rated as yes, no, unclear, or not applicable. Disagreements were resolved by consensus or third-party adjudication (P.R.M-F.).

It was necessary to adapt the original checklist for experimental ballistic studies. Accordingly, interpretative modifications were applied as follows. Sample inclusion was assessed in terms of whether biological and non-biological target materials were clearly described, including their provenance and suitability for the experimental model. Study participants and setting were defined by the firearm used, the type and lot of ammunition, and the experimental setup. Exposure measurement was evaluated as the placement of samples in the same environment as the firearm discharge. The measurement of the condition was assessed in terms of whether objective and standardized criteria were applied for the detection of GSR by micro-CT. Confounding factors and strategies to address them included potential pre-existing contamination, since micro-CT detects only Hounsfield units and cannot identify specific particles, the time elapsed before sample collection, shooting conditions (distance, caliber, ammunition type), and image acquisition noise. Results assessment referred to the presence and morphology of GSR detected by micro-CT.

### Data analysis

Data related to GSR detection by micro-CT were organized into a spreadsheet dataset and analyzed using R software (version 4.5.1; R Foundation for Statistical Computing, Vienna, Austria). The primary outcome was the mean percentage of detectable GSR, derived from quantitative micro-CT measurements. Variance estimates were derived based on the reported dispersion measures and sample sizes, allowing appropriate weighting in subsequent analyses.

### Random-effects meta-analyses

Four random-effects meta-analyses were conducted: one overall analysis pooling data across the available shooting distances (5, 15, 23, 30, and 40 cm) and three distance-specific analyses (5, 15, and 30 cm). Stratified analyses were restricted to distances represented in multiple studies, excluding the 23 and 40 cm conditions. Sensitivity analyses were also conducted by sequentially excluding the 23 and 40 cm distance conditions, individually and simultaneously, to assess their impact on the pooled estimates and heterogeneity measures. Between-study heterogeneity was modeled using a random-effects framework with restricted maximum likelihood (REML) estimation. Pooled estimates of the mean percentage of GSR detected were accompanied by τ², I², and Cochran’s Q statistics [26, 27]. Study weights were calculated as the inverse of the adjusted variance and normalized to sum to 100%. Statistical significance was assessed at a 5% level.

### Quadratic mixed-effects meta-regression

A quadratic mixed-effects meta-regression was fitted to examine the continuous relationship between shooting distance and the mean percentage of GSR detected. All available firing distances were included in this analysis, including distances represented by a single study, since the objective was to model the continuous distance-response relationship instead of deriving distance-specific pooled estimates. Analyses were conducted on the original percentage scale, which was retained throughout model fitting and prediction. The model included a random intercept for study identifier to account for within-study dependence, with remaining heterogeneity modeled at the study level. Fixed effects consisted of centered shooting distance and its quadratic term. Parameter estimation was performed using REML [26].

Uncertainty in both the fitted values and the estimated vertex of the quadratic function was quantified using 2,000 parametric bootstrap replications. Bootstrap samples were generated by drawing from a multivariate normal distribution parameterized by the REML fixed-effects estimates and their variance-covariance matrix [28]. For each replication, predicted values were recomputed and the vertex (turning point) of the curve was analytically derived from the quadratic parameters. Empirical bootstrap distributions were then used to obtain 95% confidence intervals. Results were visualized as scatter plots overlaid with the fitted quadratic curve and corresponding bootstrap confidence bands, with point sizes proportional to observational precision (inverse square root of the variance) and colors indicating sample-stage categories.

### Gaussian fits with bootstrap confidence intervals

As an exploratory complementary analysis, weighted nonlinear Gaussian models were fitted to characterize the decay pattern of GSR detection as a function of shooting distance, stratified by sample-stage category. Model fitting was performed using the Levenberg–Marquardt algorithm implemented in the nlsLM function [29, 30], with inverse-variance weighting. Parameters representing the peak detection amplitude and decay rate were estimated, and uncertainty was assessed through 1000 nonparametric bootstrap resamplings [28], yielding 95% confidence intervals. Descriptive R² values were used to summarize the agreement between observed data and the fitted Gaussian profiles. Values close to 1 indicate a close correspondence between model and data, whereas negative values indicate that the fitted curve does not reduce residual variance relative to a constant (mean-only) model.

## Results

### Study selection

**Fig. 1:**
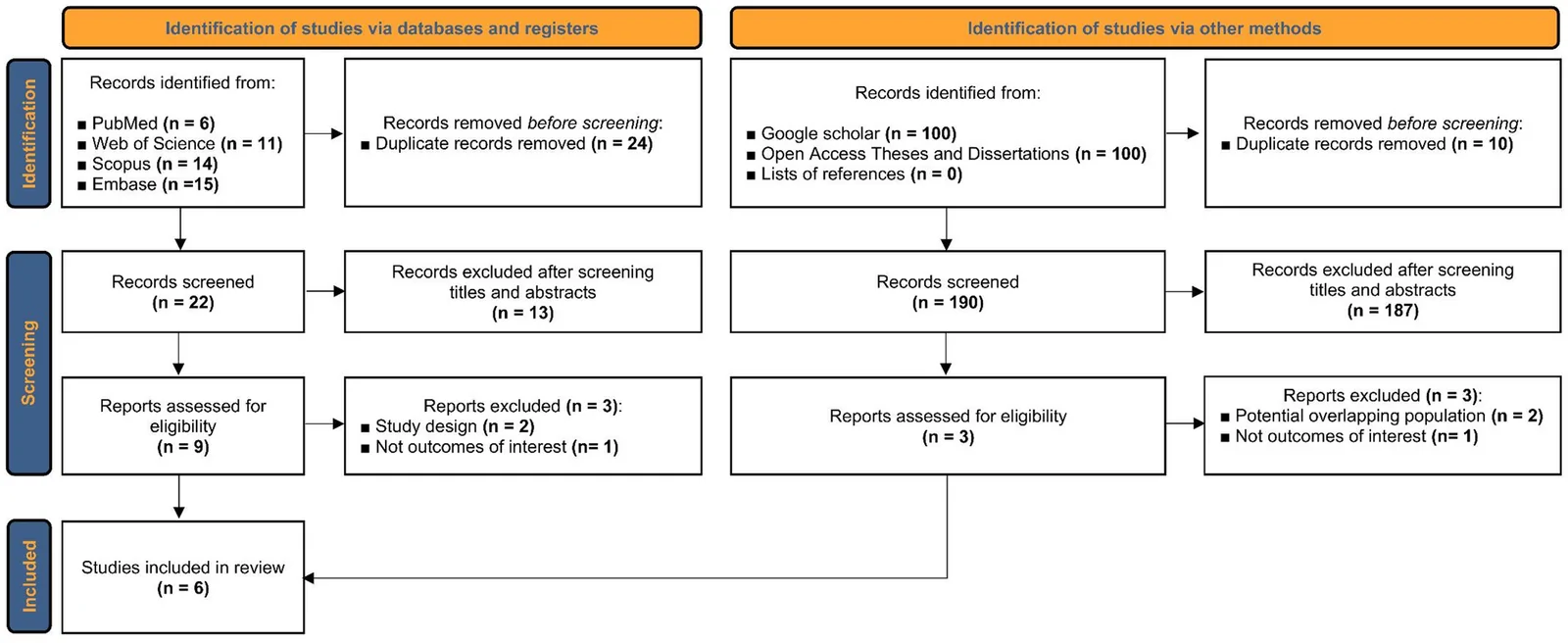
Flow diagram of study selection

The literature search identified 246 records across databases and supplementary sources. After title and abstract screening, 12 full-text articles were assessed for eligibility. Six studies were excluded due to inappropriate design (*n* = 2), absence of relevant outcomes (*n* = 2), or potential population overlap (*n* = 2). Finally, six studies met the inclusion criteria and were incorporated into the systematic review and quantitative analyses [31–36]. The study selection process is presented in Fig. 1.

### Characteristics of the included studies

All six included studies applied micro-CT to detect or characterize GSR. Five studies evaluated biological tissues obtained from human lower limbs [31–35], whereas one study analyzed non-biological textile substrates [36].

### Biological samples

All five studies involving biological substrates were conducted in Italy between 2011 and 2016 [31–35] and employed standardized human lower-limb sections obtained from surgical amputations. Each specimen measured approximately 6 cm in length and originated from male donors aged 20–50 years. Sample sizes ranged from 24 to 75 segments per study.

Experimental protocols were highly standardized across investigations. Gunshots were delivered using a semi-automatic Beretta Model 81 pistol (caliber 7.65 × 17 mm Browning, 0.32 ACP) with full metal jacket ammunition from the same manufacturing lot. The firearm was mounted on a fixed stand positioned approximately perpendicular to the target surface to ensure consistent trajectory. Depending on the study design, between 6 and 30 shots were fired per distance, with firing distances ranging from 5 to 40 cm.

**Fig. 2:**
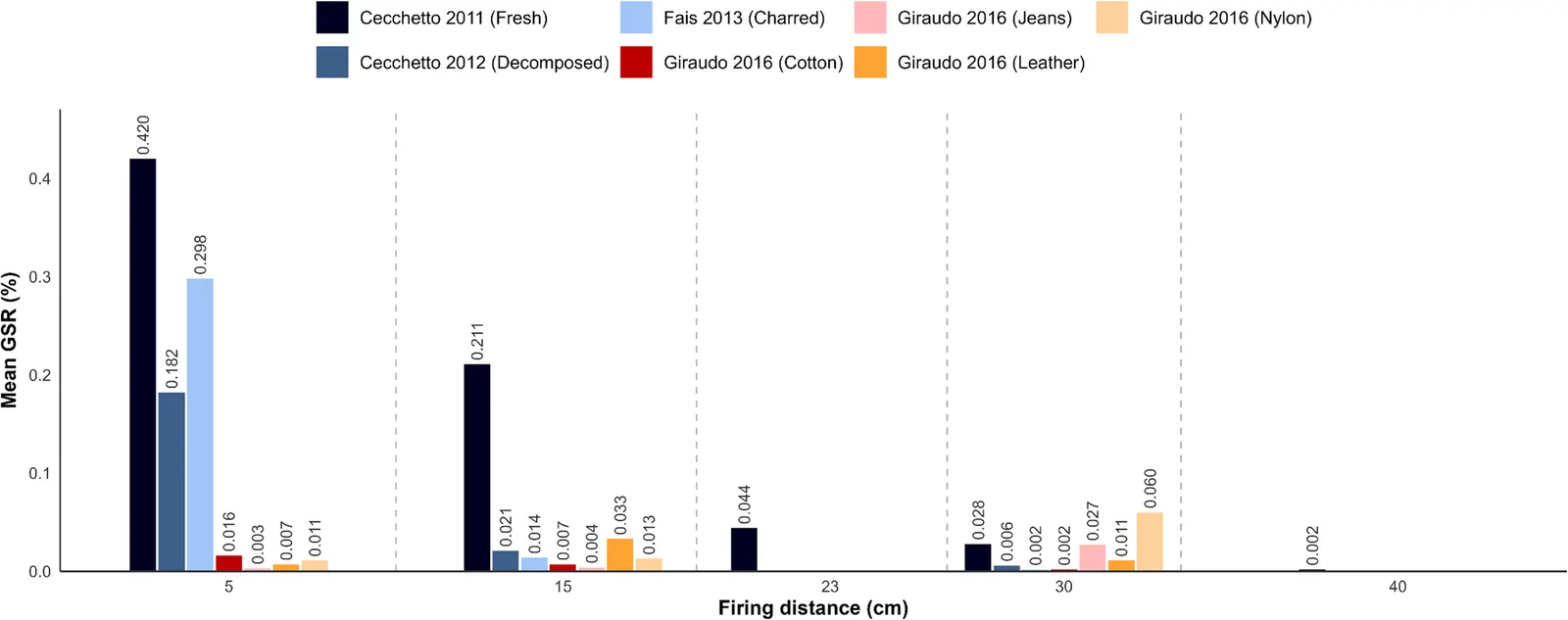
Percentage of GSR detected by micro-CT according to shooting distance in studies using biological samples

All studies employed the same high-resolution micro-CT system (Skyscan 1172) under identical acquisition settings. Image reconstruction and analysis were performed using N-Recon, CT-An, and CT-Vox software. System calibration was conducted with a water phantom, and GSR particles were identified using a radiodensity threshold exceeding 1000 Hounsfield Units (HU). Detailed technical specifications are provided in Tables 1 and 2. Across studies, the primary quantitative outcome was the proportion of GSR detected as a function of shooting distance, summarized in Fig. 2.

**Table 1:** Characteristics of the included studies

Author		Giovanni Cecchetto et al.	Giovanni Cecchetto et al.	Paolo Fais et al.	Paolo Fais et al.	Chiara Giraudo et al.	Zuzanna Brożek-Mucha et al.
**Year**		2011	2012	2013	2015	2016	2020
**Country**		Italy	Italy	Italy	Italy	Italy	Poland
**Sample**		60 sections of human legs (~ 6 cm length each)	60 sections of human legs (~ 6 cm length each)	24 (~ 6 cm length each)	30 (~ 6 cm length each)	75 (~ 6 cm length each)	6 white cotton fabric fragments (~ 40 × 40 cm)
**Sex**	**Male**	60	60	24	30	75	*NA*
	**Female**	0	0	0	0	0	*NA*
**Age range**		20–50	20–50	20–50	20–50	20–50	*NA*
**Firearm**		Semi-automatic Pistol Beretta mod. 81	Semi-automatic Pistol Beretta mod. 81	Semi-automatic Pistol Beretta mod. 81	Semi-automatic Pistol Beretta mod. 81	Semi-automatic Pistol Beretta mod. 81	Hunting rifle
**Caliber**		7.65 × 17 mm Browning (or 0.32 ACP)	7.65 × 17 mm Browning (or 0.32 ACP)	7.65 × 17 mm Browning (or 0.32 ACP)	7.65 × 17 mm Browning (or 0.32 ACP)	7.65 × 17 mm Browning (or 0.32 ACP)	12-gauge
**Bullet characteristics**		FMJ (0.32 ACP or 7.65 × 17 mm Browning SR), same lot	FMJ (0.32 ACP or 7.65 × 17 mm Browning SR), same lot	FMJ (0.32 ACP or 7.65 × 17 mm Browning SR), same lot	FMJ (0.32 ACP or 7.65 × 17 mm Browning SR), same lot	FMJ (0.32 ACP or 7.65 × 17 mm Browning SR), same lot	12/70 W8MP cartridges; lead projectile, Brenneke type, 16.5 mm, 29.5 g; plastic wad 8.3 mm
**Firearm setup**		Positioned on fixed stand, perpendicular to sample	Positioned on fixed stand, perpendicular to sample	Positioned on fixed stand, perpendicular to sample	Positioned on fixed stand, perpendicular to sample	Positioned on fixed stand, perpendicular to sample	*NR*
**Gunshots per distance**		10	20	6	30	25	1
**Firing distances (cm)**		5, 15, 23, 30, and 40	5, 15, and 30	5, 15, and 30	15	5, 15, and 30	0, 10, 20, 30, 50, and 70
**Analysis / Outcome**		Detection and characterization of GSR; 3D reconstruction of gunshot wounds; quantification of GSR percentage in relation to firing distance.	Detection and characterization of GSR by micro-CT; comparison between fresh and composed samples (15 days at open air with an average temperature of 24.5 °C and na average humidity of 64.9%).	Detection and quantification of GSR in charred samples (4 min at a temperature of 600 °C) by micro-CT; comparison with fresh controls and stab wounds.	Evaluation of the effects of different conditions (fresh, fabric-covered, water-immersed for 1 day at 28 °C, decomposed for 15 days at open air with an average temperature of 24.5 °C and humidity of 64.9%, and heat-exposed inside a wood-burning stove for 4 min at 600 °C) on GSR detection.	Analysis of the impact of different fabrics (cotton, denim, leather, and nylon), and firing distance on GSR detection using micro-CT.	Micro-CT: It was necessary to cut the fragments of cotton woven fabric to dimensions 5 × 5 cm with a bullet hole in the centre.

*NA:* Not applicable*;ACP:* Automatic Col Pistol*;FMJ: *Full Metal Jacket*; NR* Not reported

**Table 2:** Imaging acquisition, processing, and visualization methods

Author		Giovanni Cecchetto et al.	Giovanni Cecchetto et al.	Paolo Fais et al.	Paolo Fais et al.	Chiara Giraudo et al.	Zuzanna Brożek-Mucha et al.
**Year**		2011	2012	2013	2015	2016	2020
**Micro-CT model**		Skyscan 1172 h Micro-CT (Skyscan, Artselaar, Belgium)	Skyscan 1172 h Micro-CT (Skyscan, Artselaar, Belgium)	Skyscan 1172 h Micro-CT (Skyscan, Artselaar, Belgium)	Skyscan 1172 h Micro-CT (Skyscan, Artselaar, Belgium)	Skyscan 1172 h Micro-CT (Skyscan, Artselaar, Belgium)	Nanotom 180 N (Ge Sensing & Inspection Technologies phoenix-ray Gmbh, wunstorf, Germany)
**Voltage (kV)**		100	100	100	100	100	100
**Current (µA)**		100	100	100	100	100	150
**Filter**		Al, 1 mm of thickness	Al, 1 mm of thickness	Al, 1 mm of thickness	Al, 1 mm of thickness	Al, 1 mm of thickness	*NR*
**Voxel size (µm)**		13	13	13	13	13	20
**FOV (pixels)**		1280 × 1024	1280 × 1024	1280 × 1024	1280 × 1024	1280 × 1024	2300 × 2300 (Hamamatsu detector)
**Scan rotation**		360º, step 0.4º, frame averaging 2	360º, step 0.4º, frame averaging 2	360º, step 0.4º, frame averaging 2	360º, step 0.4º, frame averaging 2	360º, step 0.4º, frame averaging 2	360º in 2400 steps, frame averaging 3, image skip 1
**Scan duration**		*NR*	*NR*	45 min	*NR*	45 min	100 min (500 ms exposure)
**VOI**		1 cm x 1 cm x 3.8 mm, centered on the wound	1 cm x 1 cm x 3.8 mm, centered on the wound	1 cm x 1 cm x 3.8 mm, centered on the wound	1 cm x 1 cm x 3.8 mm, centered on the wound	1 cm x 1 cm x 3.8 mm, centered on the wound	5 × 5 cm, centered on the wound
**Software**	**Reconstruction**	N-Recon (Skyscan, Artselaar, Belgium)	N-Recon (Skyscan, Artselaar, Belgium)	N-Recon (Skyscan, Artselaar, Belgium)	N-Recon (Skyscan, Artselaar, Belgium)	N-Recon (Skyscan, Artselaar, Belgium)	DatosX 2.1.0 (GE) using Feldkamp algorithm
	**Analysis**	CT-An (Skyscan, Artselaar, Belgium)	CT-An (Skyscan, Artselaar, Belgium)	CT-An (Skyscan, Artselaar, Belgium)	CT-An (Skyscan, Artselaar, Belgium)	CT-An (Skyscan, Artselaar, Belgium)	VGStudio Max 2.1 (Volume Graphics Gmbh), and Fiji
	**3D Rendering**	CT-Vox (Skyscan, Artselaar, Belgium)	CT-Vox (Skyscan, Artselaar, Belgium)	CT-Vox (Skyscan, Artselaar, Belgium)	CT-Vox (Skyscan, Artselaar, Belgium)	CT-Vox (Skyscan, Artselaar, Belgium)	ImageJ (BoneJ plug-in)
**HU calibration**		Water phantom	Water phantom	Water phantom	Water phantom	Water phantom	*NR*
**GSR threshold (HU)**		> 1000	> 1000	> 1000	> 1000	> 1000	*NR*

*kV:* Kilovoltage*;* *µA:* microampere*;* *Al:* Aluminum*;* *NR:* Not reported*;* *µm:* micrometer*;* *FOV:* Field of View*;* *ms:* millisecond*;* *VOI:* Volume of interest*;* *HU:* Hounsfield unit

### Non-biological samples

The single study investigating non-biological substrates was conducted in Poland and published in 2020 [36]. The experimental material consisted of six standardized cotton fabric fragments measuring approximately 40 × 40 cm. Gunfire exposure was performed using a 12-gauge hunting rifle with 12/70 W8MP cartridges loaded with lead Brenneke projectiles. One shot was fired at each distance, and shooting distances ranged from direct contact (0 cm) to 70 cm (0, 10, 20, 30, 50, and 70 cm), providing a broader spatial gradient than that used in the biological models.

Micro-CT imaging was conducted using a Nanotom 180 N system. Reconstruction employed DatosX 2.1.0 with the Feldkamp algorithm, and image processing and particle analysis were performed using VGStudio Max, Fiji, and ImageJ. Detailed acquisition and reconstruction parameters are provided in Tables 1 and 2. Unlike the biological studies, which reported detection percentages, this investigation quantified GSR deposition based on particle size metrics. At contact shots (0 cm), inorganic GSR particles and aggregates ranged from 53.8 to 465.9 μm, with a mean particle size of 184.8 μm (SD = 77.8 μm). At 10 cm, a marked reduction in particle size was observed (40.0–240.0 μm), with a mean of 83.4 μm (SD = 38.0 μm). At longer distances, mean particle sizes increased again, reaching 150.8 μm (SD = 64.9 μm) at 50 cm and 186.0 μm (SD = 82.7 μm) at 70 cm. These findings indicate a non-linear relationship between shooting distance and mean GSR particle size in cotton fabric substrates.

### Risk of bias assessment

Most studies provided detailed descriptions of experimental procedures and exposure measurements. However, strategies to control potential confounding factors were rarely specified. Variability in shooting conditions was limited primarily to firing distance, with minimal exploration of alternative firearms or ammunition types. Additionally, eligibility criteria were insufficiently reported in the non-biological study.

Based on the predefined assessment tool, studies achieved high proportions of positive methodological ratings; nevertheless, important sources of uncertainty remained. Consequently, the overall risk of bias was judged as low-to-moderate, with limitations related to restricted external validity and experimental standardization. The complete assessment is presented in Table 3.

**Table 3:** Risk of bias assessment

Question	Cecchetto 2011	Cecchetto 2012	Fais 2013	Fais 2015	Giraudo 2016	Brożek-Mucha 2020	Total (%)	
Were the criteria for inclusion in the sample clearly defined?	**▲**	**▲**	**▲**	**▲**	**▲**	▬	▲	**83.3**
							▬	**16.7**
Were the study subjects and the setting described in detail?	**▲**	**▲**	**▲**	**▲**	**▲**	**▲**	▲	**100.0**
							▬	**0.0**
Was the exposure measured in a valid and reliable way?	**▲**	**▲**	**▲**	**▲**	**▲**	**▲**	▲	**100.0**
							▬	**0.0**
Were objective, standard criteria used for measurement of the condition?	**▲**	**▲**	**▲**	**▲**	**▲**	**▲**	▲	**100.0**
							▬	**0.0**
Were confounding factors identified?	**▲**	**▲**	**▲**	**▲**	**▲**	**▲**	▲	**100.0**
							▬	**0.0**
Were strategies to deal with confounding factors stated?	▬	▬	▬	▬	▬	▬	▲	**0.0**
							▬	**100.0**
Were the outcomes measured in a valid and reliable way?	**▲**	**▲**	**▲**	**▲**	**▲**	**▲**	▲	**100.0**
							▬	**0.0**
Was appropriate statistical analysis used?	**▲**	**▲**	**▲**	**▲**	**▲**	**▲**	▲	**100.0**
							▬	**0.0**
**Total (%)**	▲ **87.5**	▲ **87.5**	▲ **87.5**	▲ **87.5**	▲ **87.5**	▲ **75.0**	**▲**	**85.41**
	▬ **12.5**	▬ **12.5**	▬ **12.5**	▬ **12.5**	▬ **12.5**	▬ **25.0**	**▬**	**14.59**

▲, *Yes; ▬*,Unclear. Items were interpreted according to adaptations described in the methods section

### Qualitative and quantitative synthesis

Qualitative assessment across the included studies confirmed the capability of micro-CT for non-destructive visualization and spatial localization of GSR. Particles were consistently identified as high-contrast radiodense voxels (typically > 1000 HU), enabling three-dimensional mapping of residue clusters within the substrate volume. In biological specimens, micro-CT allowed localization of particles embedded within subcutaneous layers, whereas in textile substrates it allowed visualization of particles trapped between fibers.

All quantitative analyses were based on four studies evaluating biological substrates exposed to gunfire at multiple distances [31–33, 35]. These investigations reported mean percentages of GSR detection according to shooting distance under different experimental conditions, including fresh, decomposed, and charred tissues, as well as fabric-covered surfaces (cotton, jeans, leather, and nylon). Extracted summary measures (means, standard deviations, sample sizes, and corresponding distances) are provided in Supplementary Table 1.

### Random-effects meta-analyses

**Fig. 3:**
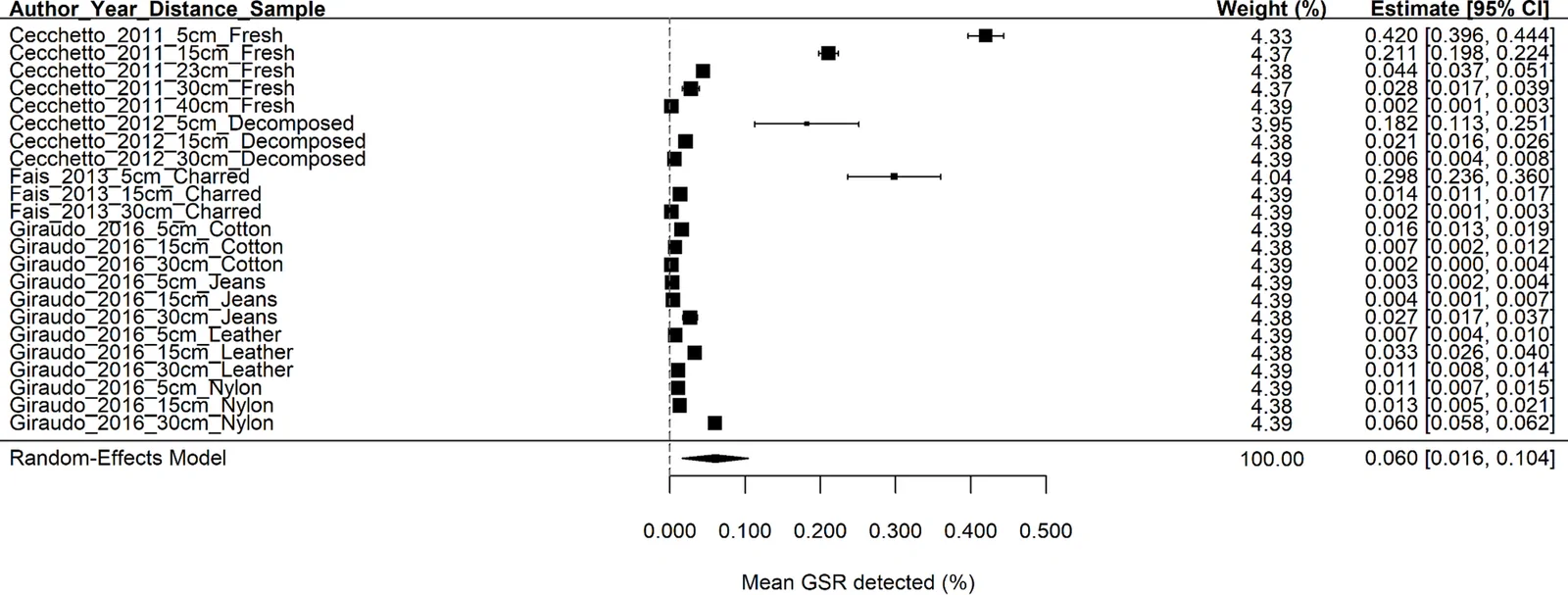
Global random-effects meta-analysis showing the estimated mean percentage of GSR detected by micro-CT, with corresponding 95% confidence intervals

**Fig. 4:**
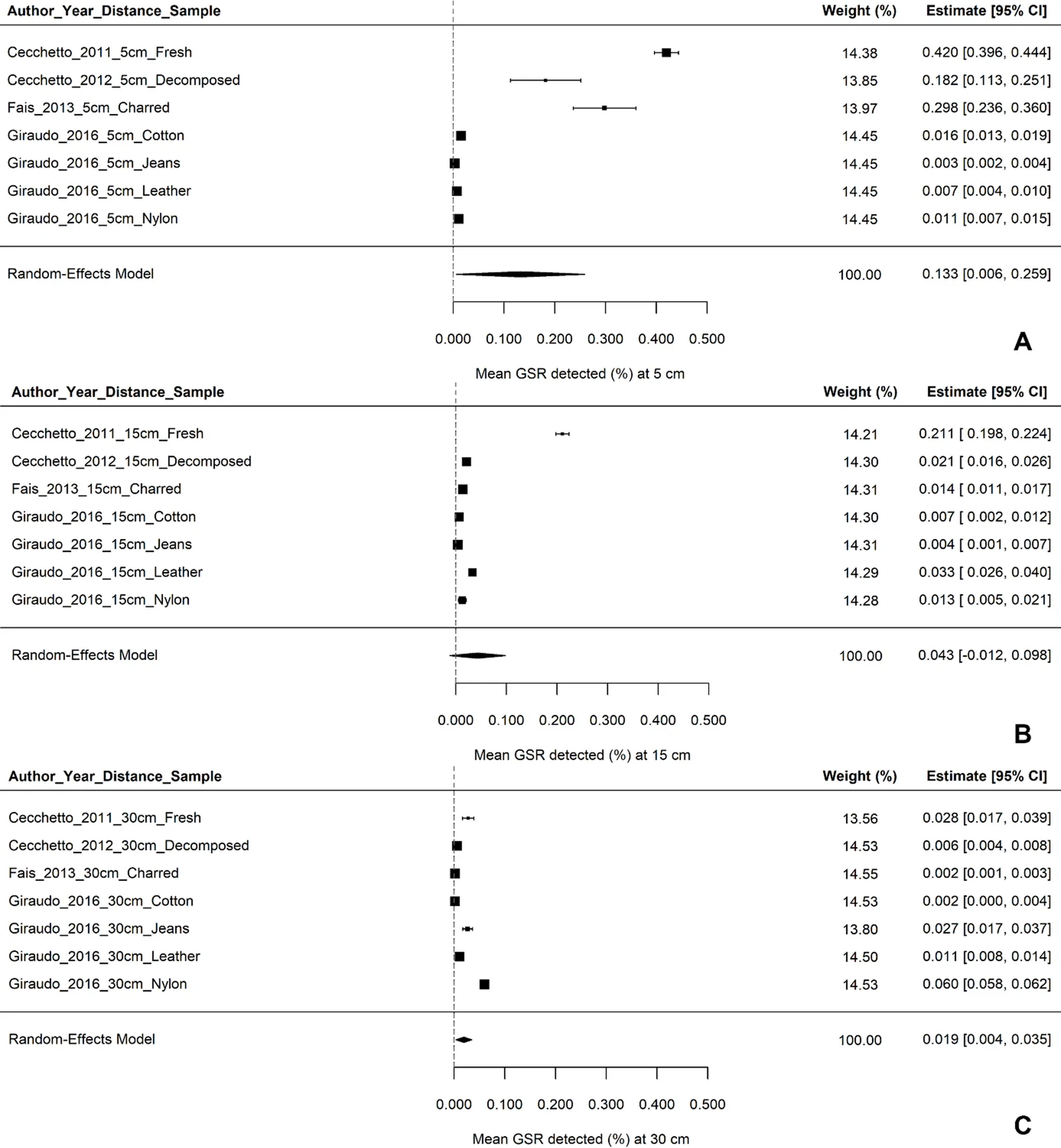
Distance-stratified random-effects meta-analyses showing the estimated mean percentages of GSR detected by micro-CT at different firing distances: (**A**) 5 cm, (**B**) 15 cm, and (**C**) 30 cm, each with corresponding 95% confidence intervals. The 23 cm and 40 cm conditions were not included because they were represented by a single study

The overall random-effects model yielded a pooled mean GSR detection rate of 0.060% (95% CI: 0.016–0.104; *p* = 0.007). Sensitivity analyses showed only minor changes in pooled estimates following sequential exclusion of the 23 cm and 40 cm distance conditions. Exclusion of the 23 cm condition resulted in a pooled estimate of 0.061% (95% CI: 0.015–0.107; *p* = 0.009), while exclusion of the 40 cm condition yielded an estimate of 0.063% (95% CI: 0.017–0.109; *p* = 0.007). Simultaneous exclusion of both distance conditions produced a pooled estimate of 0.064% (95% CI: 0.016–0.112; *p* = 0.009). Compiled sensitivity analysis results are presented in Supplementary Table S2. Distance-stratified analyses showed decreasing detection with increasing firing distance, from 0.133% at 5 cm to 0.019% at 30 cm. Effect estimates were statistically significant at 5 cm and 30 cm (*p* < 0.05) but not at 15 cm (*p* = 0.124). Between-study heterogeneity was extremely high across all models (I² > 99%), indicating substantial between-study variability. Summary estimates and heterogeneity statistics are reported in Table 4, with forest plots presented in Figs. 3 and 4.

**Table 4:** Results of the global and distance-stratified random-effects meta-analyses showing estimated mean percentages of GSR detected by micro-CT

Meta-analysis	k	Estimated mean [%, 95% CI]	*p*-value	τ	τ²	I² (%)	Cochran’s Q test Q (df)	*p*(Q)
Global	23	0.060 [0.016–0.104]	0.007	0.107	0.012	99.99	Q (22) = 6562.21	< 0.001
5 cm	7	0.133 [0.006–0.259]	0.039	0.169	0.029	99.99	Q (6) = 1404.34	< 0.001
15 cm	7	0.043 [-0.012–0.098]	0.124	0.074	0.005	99.89	Q (6) = 978.16	< 0.001
30 cm	7	0.019 [0.004–0.035]	0.016	0.021	< 0.001	99.78	Q (6) = 3628.93	< 0.001

*k:* number of observations included in each meta-analysis; Estimated mean [%, 95% CI]: mean percentage of GSR detected by micro-CT estimated by the random-effects model, with corresponding 95% confidence interval; p-value: statistical significance of the estimated overall effect; τ: estimated between-study standard deviation; τ²: estimated between-study variance; I² (%): proportion of total variability due to heterogeneity rather than chance; **Q (df) Cochran’s Q test**: statistic and degrees of freedom. p(Q): p-value associated with the heterogeneity test

### Quadratic mixed-effects meta-regression

Mixed-effects meta-regression identified a significant nonlinear association between shooting distance and GSR detection. The quadratic term for the centered distance variable was statistically significant (*p* < 0.001), while the linear term was not statistically significant (*p* = 0.286). The negative quadratic coefficient indicated a concave (downward-opening) relationship between distance and detection rate.

**Fig. 5:**
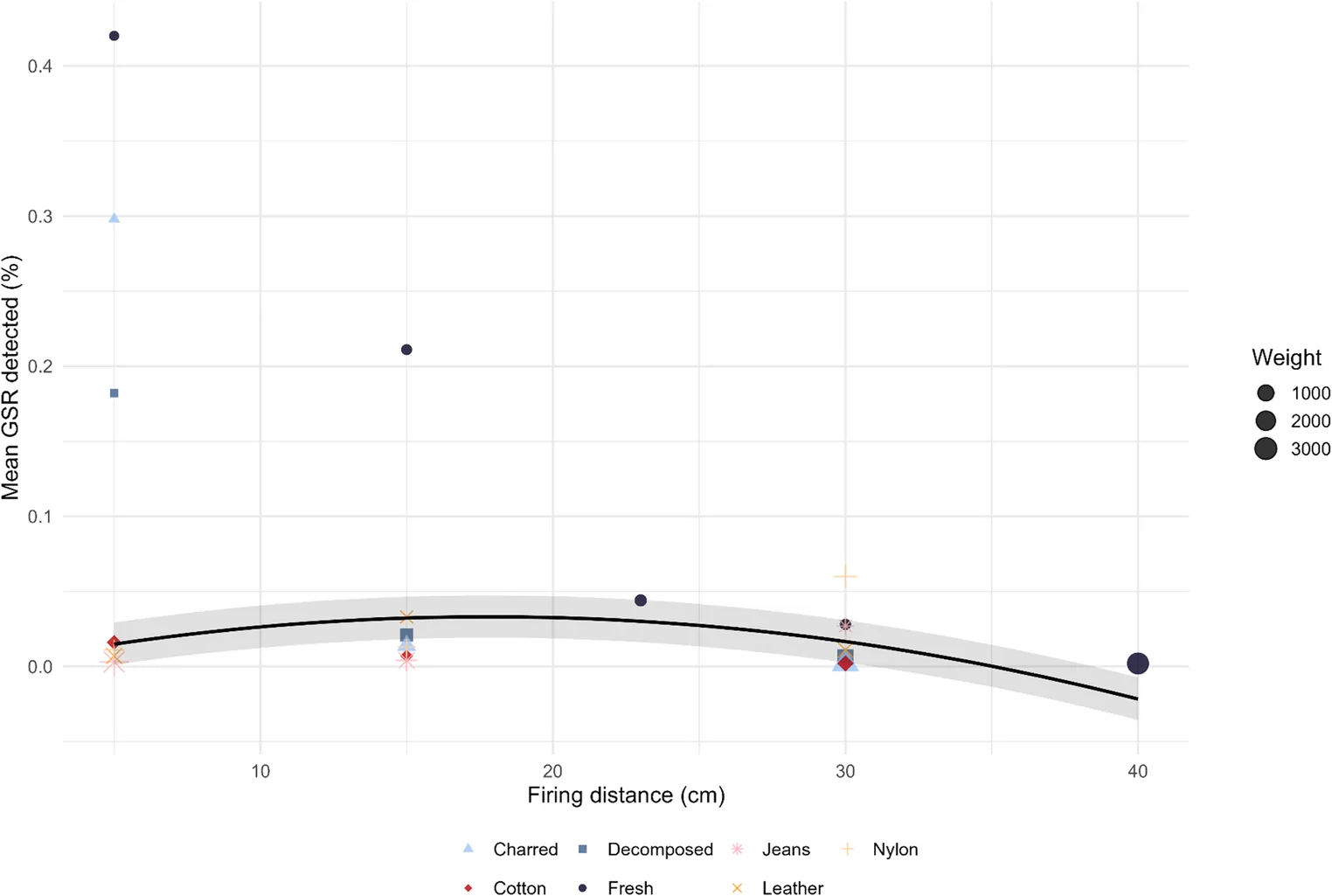
Quadratic mixed-effects meta-regression of gunshot distance versus percentage of GSR detected by micro-CT, with fitted curve and 95% confidence intervals

The intercept corresponded to a predicted baseline detection of 0.033% (*p* < 0.001). Residual heterogeneity remained extremely high (I² = 99.52%), and distance accounted for a negligible proportion of the between-study variance (pseudo-R² = 0%), consistent with the substantial variability expected in forensic sampling conditions. The fitted curve estimated a maximum predicted detection at 17.79 cm (bootstrap 95% CI: 17.47–18.10 cm). Full regression coefficients, standard errors, and confidence intervals are presented in Table 5, and model predictions are illustrated in Fig. 5.

**Table 5:** Results of the quadratic mixed-effects meta-regression examining the relationship between firing distance and the percentage of GSR detected by micro-CT

Term	Estimate (b)	Std. Error	z-value	*p*-value	τ² (total)	I² (total, %)	Pseudo-*R*² (%)	Apex [95% CI] (cm)	QE (residual heterogeneity)	QM (test of moderators)
Intercept	0.0330	0.0074	4.475	< 0.0001	0.0004	99.52	0	17.79 [17.47–18.10]	6098.76 (df = 20) *p* < 0.0001	674.808 (df = 2) *p* < 0.0001
Firing distance (centered)	–0.0000	0.0000	-1.067	0.2585						
Firing distance²	–0.0001	0.0000	–25.487	< 0.0001						

***Estimate (b)***: regression coefficient for each term in the model; **Std. Error**: standard error of the coefficient estimate; ***z-value***: test statistic for each coefficient; ***p-value***significance level of the term; **t² (total)**: estimated total between-study variance; ***I² (total***,*** %)***: proportion of total variability due to heterogeneity; **Pseudo-R²**: proportion of between-study variance explained by the model; **Apex [95% CI] (cm)**: firing distance at which the predicted GSR percentage is maximal, with 95% confidence interval from bootstrap; ***QE (Residual Heterogeneity)***: Cochran’s Q test statistic for residual heterogeneity, with degrees of freedom and p-value; ***QM (Test of Moderators)***: test statistic for the joint significance of moderators in the model, with degrees of freedom and p-value

### Gaussian fits with bootstrap confidence intervals

**Fig. 6:**
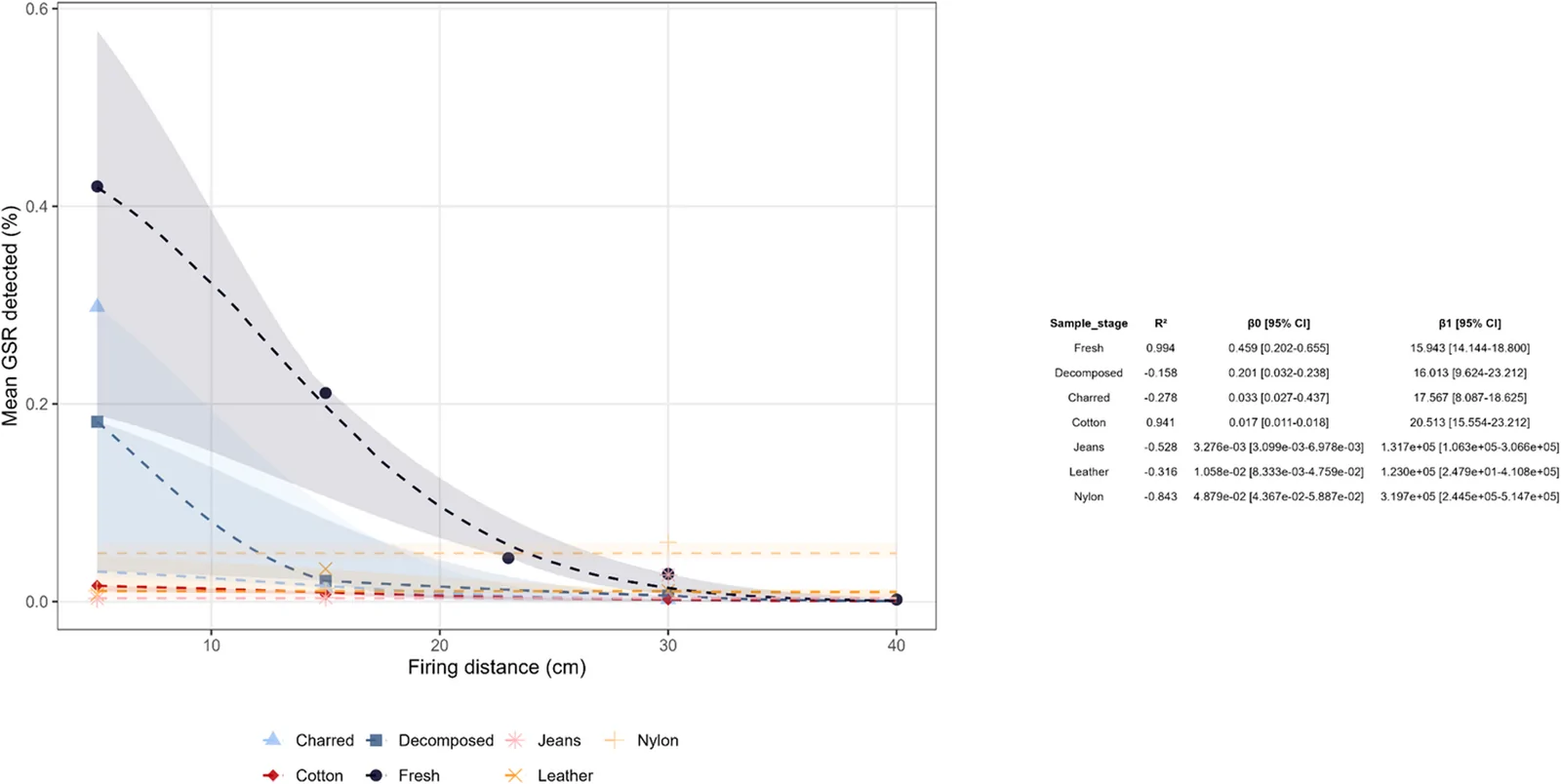
Weighted Gaussian fits of GSR detection for each sample stage showing the parameters β0 and β1 with 95 percent bootstrap confidence intervals. R² values are displayed for descriptive purposes only

Weighted Gaussian models were fitted separately for each substrate condition to describe the relationship between shooting distance and GSR detection. Parameter estimates (β₀, β₁) and bootstrap 95% confidence intervals are presented in Supplementary Table S3, with fitted curves shown in Fig. 6, where descriptive R² values are provided to indicate the degree of agreement between observed data and the fitted profiles. Fresh biological tissues exhibited the highest baseline detection, with β₀ = 0.46% (95% CI: 0.20–0.65) and a decay parameter β₁ = 15.94 cm (95% CI: 14.14–18.80). Decomposed and charred tissues showed substantially lower baseline values (β₀ = 0.03%), accompanied by larger uncertainty and wider confidence intervals for β₁. For textile substrates (cotton, jeans, leather, and nylon), baseline detection remained low (β₀ range: 0.003–0.049%), with large β₁ estimates, in some cases exceeding 100 cm, indicating minimal observable decay within the evaluated distance range. These estimates demonstrate marked variability in detection profiles across substrate conditions.

Descriptive R² values indicated a close agreement between observed data and the fitted Gaussian profiles for fresh biological tissue (R² = 0.99) and cotton (R² = 0.94). In contrast, negative R² values were observed for decomposed, charred, jeans, leather, and nylon substrates, indicating that the Gaussian model did not reduce residual variability relative to a constant model for these conditions.

## Discussion

Firearm-related violence continues to represent a major global public health and security concern and accounts for a substantial proportion of homicides worldwide [16, 37]. Within this context, forensic ballistics plays a critical role in reconstructing shooting events and establishing associations among suspects, victims, and firearms [8, 15, 38]. The analysis of gunshot residue (GSR) constitutes one of the most informative trace-based approaches within this field, as the presence, distribution, and composition of residue particles may support inferences regarding discharge occurrence, firing distance, and potential secondary transfer [18, 39–44].

GSR is composed of heterogeneous inorganic and organic fractions generated during cartridge ignition, including lead, barium, and antimony-rich particles that are relatively stable and radiodense, as well as more volatile organic residues [45, 46]. Because these particles are microscopic and irregularly distributed, analytical techniques capable of non-destructive detection, three-dimensional localization, and morphometric characterization are particularly advantageous [18, 42, 46, 47]. Within this framework, micro-CT has emerged as a complementary modality that enables volumetric visualization and quantification of radiopaque microparticles across diverse substrates [22]. Owing to its reliance on differences in X-ray attenuation coefficients expressed in Hounsfield units, micro-CT preferentially detects the inorganic, metal-rich fraction of GSR, which produces sufficient contrast for reliable identification [47, 48].

Despite these theoretical advantages, the literature has lacked a quantitative synthesis evaluating the performance of micro-CT for GSR detection and its potential application to shooting distance estimation [11, 31–36]. The present systematic review therefore sought to consolidate and critically appraise the available experimental evidence in both biological and non-biological matrices. The pooled analyses demonstrated a consistent decline in micro-CT–detectable GSR with increasing shooting distance. This inverse association was observed across all modeling strategies and aligns with the expected reduction in particle deposition density as dispersion increases.

While between-study heterogeneity was substantial, this variability likely reflects the intrinsically multifactorial nature of GSR deposition, including differences in substrate characteristics, particle behavior, local deposition dynamics, and experimental conditions. Importantly, inorganic residues remained detectable even under heterogeneous conditions, which supports the feasibility of micro-CT as a screening and localization tool. However, the magnitude of this heterogeneity also limits the interpretability of pooled estimates, which cannot be interpreted as representing a single underlying effect.

Distance-stratified meta-analyses further illustrated this pattern. Deposition levels were highest at short range (5 cm), followed by a marked reduction at intermediate distances (15 cm), and low but still measurable detection at 30 cm. These findings are consistent with classical ballistic observations indicating that particle concentration decreases rapidly with distance but that heavier fragments may still reach more distal targets. Persistent heterogeneity across strata reinforces that residue retention depends not only on distance but also on substrate texture, porosity, and surface chemistry.

The quadratic mixed-effects meta-regression provided additional evidence by modeling the continuous relationship between distance and detection probability. The significant quadratic component confirmed a nonlinear decay pattern, with an estimated vertex at approximately 17.8 cm. This value should not be interpreted as a universal physical threshold but rather as a dataset-specific summary reflecting the combined influence of emission dynamics, particle inertia, and experimental conditions. The pseudo-R² of 0% indicates that the variability observed across studies was not meaningfully explained by shooting distance alone, reinforcing the multifactorial nature of GSR deposition and the influence of additional factors such as substrate properties and local deposition dynamics.

The observed nonlinear decay in detection probability is consistent with the underlying physical mechanisms governing GSR generation and transport [49]. During discharge, primer detonation and propellant combustion produce a heterogeneous spectrum of particles varying in mass, density, and thermal state, including metallic fragments, condensed vapors, and agglomerates [18]. These particles are subsequently transported by the combined effects of the expanding gas plume, air resistance, gravity, and local turbulence [47, 50]. Consequently, dispersion is not strictly monotonic but results from the interaction between ballistic inertia and aerodynamic forces.

Heavier and denser particles exhibit greater momentum and tend to maintain trajectories closer to the projectile axis, preferentially depositing along the line of fire [34, 36, 51]. Under favorable conditions, such particles may reach intermediate or even relatively distant targets with limited angular deviation. In contrast, lighter particles are readily deflected by muzzle turbulence, vortical flows, and environmental air currents, producing broader and less predictable spatial distributions [18]. Additional factors, including firearm design, barrel length, ammunition composition, gas dynamics, and environmental conditions, further modulate these patterns, explaining why deposition profiles vary substantially across experimental setups [18, 52–54]. This physical complexity provides a mechanistic explanation for both the curved distance–detection relationship and the substantial between-study heterogeneity observed in the meta-analyses.

Within this framework, Gaussian functions offer a reasonable phenomenological approximation of deposition behavior, capturing a central high-concentration region surrounded by progressive stochastic dispersion [31, 51]. The apex of these curves corresponds to the point of maximum particle deposition and has been reported to range from a few centimeters to distances exceeding 10 m, depending on firearm characteristics, ammunition type, gas dynamics, target properties, and environmental conditions [51, 55, 56]. The fitted curves obtained in the present study yielded substrate-specific profiles that reflected differences in particle retention and detectability. Fresh biological tissues exhibited higher initial loads followed by rapid decay with increasing distance, whereas decomposed or charred substrates showed attenuated and more uncertain profiles. Synthetic materials frequently demonstrated reduced retention and flatter curves, suggesting limited adherence of microparticles. These findings indicate that detectability depends not only on emission and transport dynamics but also on the physicochemical properties of the receiving surface, including porosity, roughness, moisture content, and structural integrity.

The interpretative value of these Gaussian models varied substantially according to substrate type. High R² values for fresh biological tissue and cotton indicate that Gaussian functions adequately described the data under relatively homogeneous surface conditions, while negative R² values observed for substrates such as jeans, leather, and decomposed tissues indicate no improvement relative to a constant mean baseline or a null (mean-only) model. These findings suggest that the observed signal is largely dominated by stochastic variability and substrate-related effects, including surface porosity, texture-dependent particle loss, and structural degradation. In these cases, the Gaussian formulation represents only a limited descriptive approximation and underscores the difficulty of modeling residue behavior across heterogeneous forensic matrices.

From a practical forensic perspective, these results support the role of micro-CT primarily as a non-destructive screening and localization technique. The method enables rapid three-dimensional mapping and segmentation of radiodense microparticles, which may guide targeted confirmatory analyses and preserve sample integrity for subsequent testing. However, because image contrast is based exclusively on X-ray attenuation, micro-CT does not provide elemental specificity. Radiodense voxels may correspond to GSR particles but may also represent unrelated dense contaminants or environmental debris. Moreover, although documented contamination or secondary transfer events are relatively uncommon, the possibility of exogenous particle deposition further underscores that detected residues must always be interpreted within the broader ballistic and situational context rather than in isolation [43, 44].

In operational terms, micro-CT should be understood as an additional tool within the forensic workflow and not as a confirmatory or stand-alone method for GSR identification. Its primary utility lies in the rapid volumetric detection of radiodense structures (typically above 1000 HU), enabling the identification of regions of interest that may contain potential residue accumulations. Thus, micro-CT functions as a triage tool for analytical prioritization, supporting the selection of targeted areas for subsequent SEM/EDS examination. However, this sensitivity is not accompanied by chemical specificity, and therefore the method is inherently prone to non-specific detection of other high-density materials, including environmental contaminants and structurally similar artefacts. As a consequence, micro-CT may generate false-positive signals when interpreted in isolation. Accordingly, its role is restricted to preliminary localization and prioritization of suspect regions for subsequent analytical validation, without allowing direct inference of GSR presence. For these reasons, micro-CT findings should be interpreted alongside confirmatory analytical methods, particularly SEM/EDS, when definitive elemental characterization is required.

The principal limitation of this review is the limited and methodologically homogeneous evidence base. Only six studies satisfied the eligibility criteria, most with small sample sizes and substantial imbalance in design. Notably, five originated from the same research group and relied on highly similar experimental conditions, including firearms, calibers, ammunition lots, and micro-CT acquisition protocols. This concentration of evidence within a single research group affects the interpretation of the findings in several ways. It limits external validity, reducing extrapolation to other firearms, ammunition types, substrates, and environmental conditions. It also reduces the independence of observations across studies, since shared protocols and experimental assumptions may produce correlated outcomes. In addition, study-specific characteristics may have exerted a stronger influence on the observed effects than broader generalizable patterns. Therefore, the available evidence is better understood as reflecting a single-laboratory framework instead of an independent body of literature. Consequently, the pooled estimates derived from the meta-analyses, meta-regression, and Gaussian modeling should be interpreted primarily as descriptors of this constrained setting, not as broadly applicable parameters. The absence of independent studies from other research groups further reinforces this limitation.

An additional limitation concerns the uneven distribution of shooting distances across studies. The 23 and 40 cm conditions were represented by only a single study each, limiting the statistical representation of these distances within the pooled dataset. Sensitivity analyses demonstrated that exclusion of these conditions did not substantially alter the pooled estimates or statistical significance of the overall meta-analysis, supporting the robustness of the aggregated findings. Nevertheless, the limited availability of independent observations at intermediate and longer distances restricts the precision of continuous modeling approaches and highlights the importance of additional studies with broader and more balanced distance distributions.

Despite these limitations, the quantitative syntheses and modeling approaches consistently demonstrate that micro-CT is capable of detecting inorganic GSR particles across a range of substrates and distances, with detection probability decreasing as shooting distance increases. The quadratic meta-regression provides an overall representation of this nonlinear relationship within the analyzed dataset, whereas stratified and Gaussian models highlight the influence of substrate-specific retention mechanisms.

The extremely high residual heterogeneity (I² > 99%) indicates that the aggregated estimates were strongly influenced by methodological and structural variability and should not be interpreted as representing a single consistent effect across studies. Consequently, summary measures must be interpreted with caution, as they primarily reflect the dispersion of study-specific scenarios instead of a stable overall relationship. These findings further indicate that shooting distance alone explains little of the observed variability, emphasizing the multifactorial behavior of GSR deposition.

## Conclusion

This systematic review and meta-analysis provide the first quantitative synthesis of the application of micro-computed tomography for the detection and characterization of gunshot residue in forensic contexts. Across experimental models, micro-CT consistently identified radiodense inorganic residues and demonstrated a progressive decline in detectable deposition with increasing shooting distance, supporting the biological and physical plausibility of its use as a spatial mapping and screening tool. However, the available evidence base remains limited in size, methodologically homogeneous, and characterized by substantial between-study heterogeneity, with shooting distance explaining only a small proportion of the observed variability. These findings restrict the precision and generalizability of pooled estimates and preclude reliable distance prediction based solely on micro-CT–derived metrics.

Consequently, micro-CT should currently be interpreted as a complementary, non-destructive imaging modality that facilitates three-dimensional localization of potential residues and guides targeted confirmatory analyses rather than serving as a standalone method for elemental identification or definitive shooting-distance estimation. Future research should prioritize independent replication under diverse ballistic conditions, standardized acquisition and segmentation protocols, and integration with compositional techniques, particularly SEM/EDS as the primary confirmatory framework, to establish robust performance benchmarks and define the operational limits of micro-CT within forensic ballistic investigations.

## Supplementary Information

Below is the link to the electronic supplementary material.

Supplementary Material 1
