# Supplementary material for: Micro-computed tomography for gunshot residue detection: A systematic review and meta-analysis of distance-dependent deposition patterns
Source: Int J Legal Med. 2026 Jun 22;140(5):2907–22. doi: 10.1007/s00414-026-03879-x (PMC13499849; doi:10.1007/s00414-026-03879-x)
Supplement: Supplementary file 1 [file 414_2026_3879_MOESM1_ESM.docx]

**Micro-computed tomography for gunshot residue detection: A systematic review and meta-analysis of distance-dependent deposition patterns**

**Supplementary information**

**Table S1** Quantitative dataset used for the meta-analyses, meta-regression, and Gaussian fitting.

| **Author** | **Year** | **Study_ID** | **Firing_distance_cm** | **Sample_stage** | **n** | **Mean_percent** | **SD_percent** | **Minimum** | **Maximum** |
| --- | --- | --- | --- | --- | --- | --- | --- | --- | --- |
| Cecchetto | 2011 | Cecchetto2011_fresh | 5 | Fresh | 10 | 0.42 | 0.038 | 0.37 | 0.47 |
| Cecchetto | 2011 | Cecchetto2011_fresh | 15 | Fresh | 10 | 0.211 | 0.021 | 0.18 | 0.25 |
| Cecchetto | 2011 | Cecchetto2011_fresh | 23 | Fresh | 10 | 0.044 | 0.011 | 0.030 | 0.060 |
| Cecchetto | 2011 | Cecchetto2011_fresh | 30 | Fresh | 10 | 0.028 | 0.018 | 0.01 | 0.05 |
| Cecchetto | 2011 | Cecchetto2011_fresh | 40 | Fresh | 10 | 0.002 | 0.001 | 0.001 | 0.003 |
| Cecchetto | 2012 | Cecchetto2012_decomposed | 5 | Decomposed | 10 | 0.182 | 0.112 | 0.064 | 0.385 |
| Cecchetto | 2012 | Cecchetto2012_decomposed | 15 | Decomposed | 10 | 0.021 | 0.008 | 0.009 | 0.04 |
| Cecchetto | 2012 | Cecchetto2012_decomposed | 30 | Decomposed | 10 | 0.006 | 0.003 | 0.002 | 0.011 |
| Fais | 2013 | Fais2013_charred | 5 | Charred | 6 | 0.298 | 0.077 | 0.16 | 0.38 |
| Fais | 2013 | Fais2013_charred | 15 | Charred | 6 | 0.014 | 0.004 | 0.011 | 0.019 |
| Fais | 2013 | Fais2013_charred | 30 | Charred | 6 | 0.002 | 0.001 | 0.002 | 0.003 |
| Giraudo | 2016 | Giraudo2016_cotton | 5 | Cotton | 5 | 0.016 | 0.003 | NA | NA |
| Giraudo | 2016 | Giraudo2016_cotton | 15 | Cotton | 5 | 0.007 | 0.006 | NA | NA |
| Giraudo | 2016 | Giraudo2016_cotton | 30 | Cotton | 5 | 0.002 | 0.002 | NA | NA |
| Giraudo | 2016 | Giraudo2016_jeans | 5 | Jeans | 5 | 0.003 | 0.001 | NA | NA |
| Giraudo | 2016 | Giraudo2016_jeans | 15 | Jeans | 5 | 0.004 | 0.003 | NA | NA |
| Giraudo | 2016 | Giraudo2016_jeans | 30 | Jeans | 5 | 0.027 | 0.011 | NA | NA |
| Giraudo | 2016 | Giraudo2016_leather | 5 | Leather | 5 | 0.007 | 0.003 | NA | NA |
| Giraudo | 2016 | Giraudo2016_leather | 15 | Leather | 5 | 0.033 | 0.008 | NA | NA |
| Giraudo | 2016 | Giraudo2016_leather | 30 | Leather | 5 | 0.011 | 0.003 | NA | NA |
| Giraudo | 2016 | Giraudo2016_nylon | 5 | Nylon | 5 | 0.011 | 0.004 | NA | NA |
| Giraudo | 2016 | Giraudo2016_nylon | 15 | Nylon | 5 | 0.013 | 0.009 | NA | NA |
| Giraudo | 2016 | Giraudo2016_nylon | 30 | Nylon | 5 | 0.06 | 0.002 | NA | NA |

*NA: Not applicable.*

**Table S2** Sensitivity analyses evaluating the stability of the overall random-effects meta-analysis after sequential exclusion of the 23 cm and 40 cm distance conditions.

| **Model** | **k** | **Estimated mean  [%, 95% CI]** | **p-value** | **t²** | **I² (%)** |
| --- | --- | --- | --- | --- | --- |
| Main analysis | 23 | 0.060 [0.016 - 0.104] | 0.007 | 0.012 | 99.99 |
| Sensitivity A (removing only 23 cm) | 22 | 0.061 [0.015 - 0.107] | 0.009 | 0.012 | 99.99 |
| Sensitivity B (removing only 40 cm) | 22 | 0.063 [0.017 - 0.109] | 0.007 | 0.012 | 99.99 |
| Sensitivity C (removing 23 and 40 cm) | 21 | 0.064 [0.016 - 0.112] | 0.009 | 0.013 | 99.99 |

***k:*** *number of observations included in each meta-analysis;* ***Estimated mean [%, 95% CI]:*** *mean percentage of GSR detected by micro-CT estimated by the random-effects model, with corresponding 95% confidence interval;* ***p-value:*** *statistical significance of the estimated overall effect;* ***τ²:*** *estimated between-study variance;* ***I² (%):*** *proportion of total variability due to heterogeneity rather than chance.*

**Table S3** Gaussian fit parameters of GSR detection percentage by sample stage*.*

| **Sample stage** | **Parameter** | **Estimate** | **Std. Error** | **t-value** | **p-value** | **Bootstrap median** | **Bootstrap 95% CI** | **Residual SE** | **Iterations** | **R²** |
| --- | --- | --- | --- | --- | --- | --- | --- | --- | --- | --- |
| Fresh | β0 | 0.4708 | 0.049 | 9.701 | 0.0023 | 0.4595 | [0.202 — 0.655] | 3.82 | 5 | 0.9939 |
| Fresh | β1 | 15.933 | 0.844 | 18.875 | 0.0003 | 15.943 | [14.144 — 18.800] | 3.82 | 5 | 0.9939 |
| Decomposed | β0 | 0.0352 | 0.024 | 1.494 | 0.3760 | 0.201 | [0.032 — 0.238] | 4.24 | 6 | - 0.1579 |
| Decomposed | β1 | 22.352 | 6.899 | 3.240 | 0.1910 | 16.013 | [9.624 — 23.212] | 4.24 | 6 | - 0.1579 |
| Charred | β0 | 0.033 | 0.044 | 0.744 | 0.5930 | 0.033 | [0.027 — 0.437] | 8.61 | 7 | - 0.2776 |
| Charred | β1 | 17.567 | 8.682 | 2.025 | 0.2920 | 17.567 | [8.087 — 18.625] | 8.61 | 7 | - 0.2776 |
| Cotton | β0 | 0.017 | 0.001 | 11.383 | 0.0558 | 0.017 | [0.011 — 0.018] | 0.99 | 7 | 0.9410 |
| Cotton | β1 | 19.711 | 2.232 | 8.831 | 0.0718 | 20.513 | [15.554 — 23.212] | 0.99 | 7 | 0.9410 |
| Jeans | β0 | 0.003 | 0.002 | 1.515 | 0.3710 | 0.003 | [0.003 — 0.007] | 4.89 | 28 | - 0.5282 |
| Jeans | β1 | 1.21 × 10⁵ | 5.33 × 10¹² | 0.000 | 1.000 | 1.31 × 10⁵ | [1.1 × 10⁵ — 3.07 × 10⁵] | 4.89 | 28 | - 0.5282 |
| Leather | β0 | 0.011 | 0.008 | 1.235 | 0.4330 | 0.011 | [0.008 — 0.048] | 6.82 | 32 | - 0.3158 |
| Leather | β1 | 2.72 × 10⁵ | 1.47 × 10¹³ | 0.000 | 1.000 | 1.23 × 10⁵ | [24.787 — 4.11 × 10⁵] | 6.82 | 32 | - 0.3158 |
| Nylon | β0 | 0.0488 | 0.0651 | 0.749 | 0.591 | 0.049 | [0.044— 0.059] | 26.12 | 34 | - 0.8428 |
| Nylon | β1 | 2.45 × 10⁵ | 1.27 × 10¹³ | 0.000 | 1.000 | 3.19 × 10⁵ | [2.44 × 10⁵ — 5.14 × 10⁵] | 26.12 | 34 | - 0.8428 |

***β0:*** *maximum amplitude;* ***β1:*** *scale parameter controlling the rate of distance-dependent decay;* ***Estimate:*** *parameter value obtained from the weighted nonlinear Gaussian fit;* ***Std. Error:*** *standard error of the estimated parameter, reflecting the uncertainty of the fit;* ***t-value:*** *t-statistic for the parameter (Estimate ÷ Std. Error);* ***p-value:*** *significance level associated with the parameter test;* ***Bootstrap median:*** *median parameter value obtained from 1000 nonparametric bootstrap resamplings;* ***Bootstrap 95% CI:*** *95% confidence interval from the bootstrap, presented in brackets;* ***Bootstrap success:*** *number of bootstrap resamplings that successfully converged;* ***Residual SE:*** *residual standard error of the fit, indicating the dispersion of data around the fitted curve;* ***Iterations:*** *number of iterations required for convergence of the Levenberg–Marquardt algorithm;* ***R²****: coefficient of determination, expressing the proportion of variance explained by the fitted model relative to a null model based on the mean.* ***Note 1:*** *very high β1 values, such 10^5^, indicate practically no detectable decay in GSR percentage across the analyzed distance range.* ***Note 2:*** *Negative R² values indicate that the fitted Gaussian model did not improve data description relative to the null (mean-only) model, reflecting the absence of a detectable distance-dependent decay pattern within the evaluated range*
